# First-Principles Study on the Stability, Site Preference, Electronic Structure and Magnetism of Alloyed Fe_3_B with Ni_3_P-Type Structure

**DOI:** 10.3390/ma15175990

**Published:** 2022-08-30

**Authors:** Xiang Wei, Zhiguo Chen, Lingnan Kong, Jiwen Wu, Haichou Zhang

**Affiliations:** 1 Department of Materials Engineering, Hunan University of Humanities, Science and Technology, Loudi 417000, China; 2 School of Materials Science and Engineering, Central South University, Changsha 410083, China

**Keywords:** Fe_3_B, first-principles calculation, site preference, stability, magnetic properties

## Abstract

First-principles calculations were performed to investigate the site preference of alloying elements, and the effect of alloying elements on stability, electronic structure and magnetism of Ni_3_P-type Fe_3_B. The calculated energies suggested that all studied compounds are thermodynamically stable while it is relatively difficult to synthesize the (Fe_2.875_,Cu_0.125_)B, (Fe_2.875_,W_0.125_)B and (Fe_2.875_,Nb_0.125_)B. The (Fe_2.875_,W_0.125_)B is the most stable compound from the view of cohesive energy. Mn element prefers to occupy the Fe2 site, whereas the others are more likely to reside in the Fe1 site. It can be found from the electronic structures that the DOSs of both Fe_3_B and alloyed Fe_3_B are dominated by Fe-d states, and all the compounds mainly contain Fe-B covalent bond, Fe-Fe covalent bond and Fe-Fe metallic bond. Based on the magnetic moments (Ms) results, it can be known that the Fe_3_B, (Fe_2.875_,Mn_0.125_)B, (Fe_2.875_,Co_0.125_)B, (Fe_2.875_,Ni_0.125_)B and (Fe_2.875_,Cu_0.125_)B are ferromagnetic compounds, whereas the others are ferrimagnetic compounds. Only Mn and Co are able to enhance the magnetism of Fe_3_B. Moreover, Mn is the most favorable candidate for improving the magnetic properties of Fe_3_B among the alloying elements. These results can be used to guide the composition design and performance optimization of magnetic materials containing Fe_3_B with Ni_3_P-type structure.

## 1. Introduction

Magnetic materials play a major role in improving the performance of devices in the field of energy applications, data storage, refrigeration technologies, etc. [1]. Metastable Fe_3_B is an attractive magnetic compound due to its large saturation magnetization and reasonably strong magnetic anisotropy. In particular, it appears in various important magnetic materials, such as nanocomposite permanent magnets (e.g., Fe_3_B/Nd_2_Fe_14_B) [2,3,4,5] and Fe-B-based amorphous and nanocrystalline soft magnetic alloys [6,7,8,9]. For the nanocomposite permanent magnets, Fe_3_B, as the main phase and providing large saturation magnetization, interacts with hard magnetic phase (e.g., Nd_2_Fe_14_B) by exchange-coupling interaction, achieving large energy production. Whereas, in the latter case, nanocrystalline Fe_3_B precipitated from liquid phase or amorphous precursor is generally undesirable, mainly because it is magnetically harder than Fe-based solid solution such as α-(Fe, Si) and α-(Fe, Co, Ni). Further, to improve the magnetic properties, alloying has been widely investigated and used [4,7,10,11,12,13,14,15,16,17,18,19,20,21,22,23,24,25,26,27]. It was found that three effects can be achieved by alloying elements to affect the magnetic properties of these materials, as follows: (1) partitioning into phases and directly modifying their magnetic properties; (2) changing phase stability further to affect crystallization behaviors, and consequently influencing the size, distribution, content, and type of phases and (3) accelerating nucleation and/or suppressing the growth of phases by segregation. Numerous experimental studies have been conducted. Results show that Cr [4,10], Co [11], Mn [12], V [13], W [14] and Mo [15,16] are closely related to the former two cases, whereas Cu [17,18,19], Nb [7,20,21] and Zr [20] more easily generate segregation due to their lower solubility. It has been reported that the partial substitution of Fe by Cr probably improves the stability of Fe_3_B [10], while Cr reduces its magnetic moments [23,24,26]. Yang et al. [11] suggested that Co suppresses the crystallization of Fe_3_B, but the effect of Co on its hyperfine field (HF) in three inequivalent Fe sites is negligible. Rajasekhar et al. [12] pointed out that when Mn incorporates into Fe_3_B by replacing partial Fe, the HF values in all three Fe sites decrease linearly with increasing Mn concentration, implying that site preference is negligible for the occupation of Mn among these sites. Although alloyed Fe_3_B has significant impacts on the magnetic properties of those materials, previous research [10,13,15,22,23,24,25,26,27] mainly focused on the effects of alloying elements on crystallization behaviors, microstructures and macroscopic magnetic properties. As mentioned above, only a few works have been done involving the stability and magnetism of alloyed Fe_3_B because of the limitation of experiments. On the other hand, to the best of our knowledge, theoretical study—an effective way to predict the stability and magnetism of compounds—is missing in terms of alloyed Fe_3_B.

Fe_3_B has three isomers, namely, the Ni_3_P-type Fe_3_B with body-centered-tetragonal structure (space group: I-4), the Ti_3_P-type Fe_3_B with body-centered-tetragonal structure (space group: P4_2_/n) and the Fe_3_C-type Fe_3_B with orthorhombic structure (space group: Pnma). According to the available literature, it can be concluded that (1) pure Ni_3_P-type Fe_3_B [28,29] and Fe_3_C-type Fe_3_B [28,29,30,31,32,33,34] have been investigated by theoretical calculations, whereas the study of Ti_3_P-type Fe_3_B is missing due to the lack of atomic positional parameters; (2) theoretical [32] and experimental [35] results confirmed that the Fe_3_C-type Fe_3_B is thermodynamically more stable than the Ni_3_P-type Fe_3_B, but the relative thermodynamic stability of three Fe_3_B is still unknown because Ni_3_P-type Fe_3_B and Ti_3_P-type Fe_3_B generally are not distinguished clearly by experiments [36] and (3) their formation greatly depends on the composition of alloys [10,11,13,14,16,20,23,37]. Therefore, it is meaningful to continuously focus on these topics in order to improve our understanding of the properties of Fe_3_B and alloyed Fe_3_B, and to efficiently guide the composition design and performance optimization of Fe_3_B-containing magnetic materials. The aim of this work is to explore the site preference of alloying elements, as well as the effect of alloying elements on stability, electronic structure and magnetism of alloyed Fe_3_B with Ni_3_P-type structure at 0 K via first-principles calculations.

## 2. Computational Details

**Figure 1:**
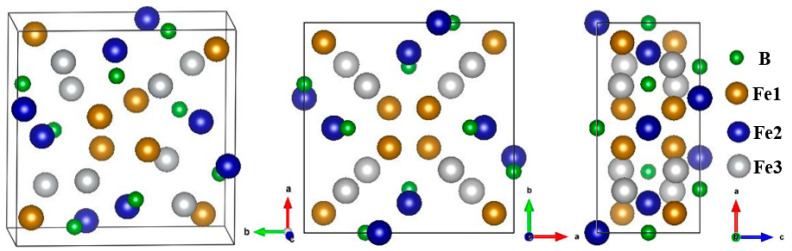
Crystal structure of Ni_3_P-type Fe_3_B.

The Ni_3_P-type Fe_3_B has a body-centered-tetragonal structure with a space group of I-4. Each unit cell contains 24 Fe atoms and 8 B atoms; that is, each cell consists of 8 Fe_3_B formula units. B atoms only have one Wyckoff site 8 g (0.288 0.046 0.491), whereas Fe atoms have three different Wyckoff sites, which are Fe1 (8 g (0.078 0.111 0.244)), Fe2 (8 g (0.364 0.031 0.988)) and Fe3 (8 g (0.166 0.220 0.751)), respectively, with equal quantities. The crystal structure of Ni_3_P-type Fe_3_B is shown in Figure 1. To obtain alloyed Fe_3_B, in the unit cell one Fe atom (Fe1 or Fe2 or Fe3) was replaced by alloying elements M (M = Ti, V, Cr, Mn, Co, Ni, Cu, Mo, W or Nb) to generate alloyed borides Fe_23_M_1_B_8_, namely, (Fe_2.875_, M_0.125_)B. All calculations were carried out by Vienna ab initio simulation package (VASP) based on density functional theory (DFT) [38]. The interactions between valence electrons and ionic cores were treated by projector-augmented wave (PAW) pseudopotential [39], where for B a standard version of PAW pseudopotential was used, and an extended M_pv version was applied for all alloying elements M. The generalized gradient approximation (GGA) method in the scheme of Perdew–Burke–Ernzerhof (PBE) was used to deal with exchange-correlation potentials [40]. The Monkhorst–Pack method was used to generate a k-points mesh in the first irreducible Brillouin zone (BZ). After extensive convergence tests, a cutoff energy of 550 eV and a 5 × 5 × 11 k-points mesh were determined for all calculations. The convergence criteria were as follows: for electronic relaxation, the maximum energy change was set to 1 × 10^−7^ eV/atom, and for ion relaxation, the maximum force acting on each atom was set to 0.001 eV/Å. In addition, spin polarization was considered for all the calculations.

## 3. Results and Discussion

### 3.1. Geometry Optimization

**Figure 2:**
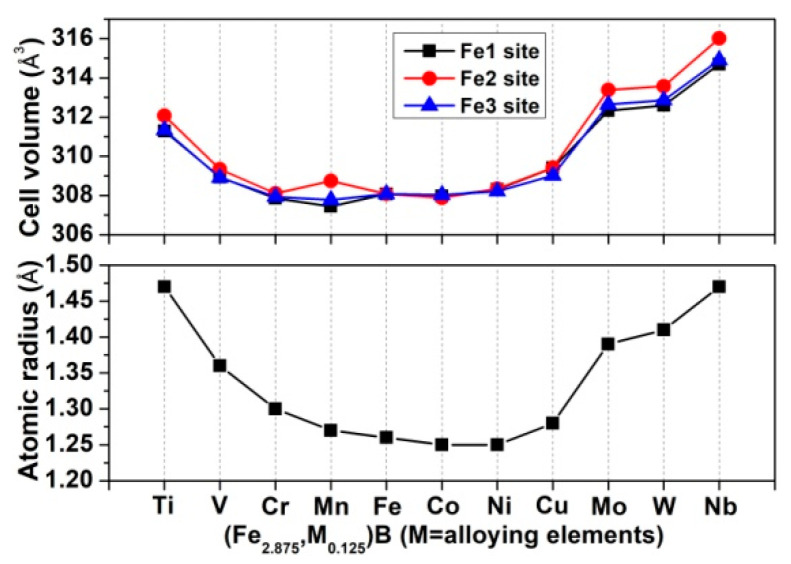
Cell volume of Fe_3_B and alloyed Fe_3_B and atomic radius of Fe and alloying elements.

**Figure 3:**
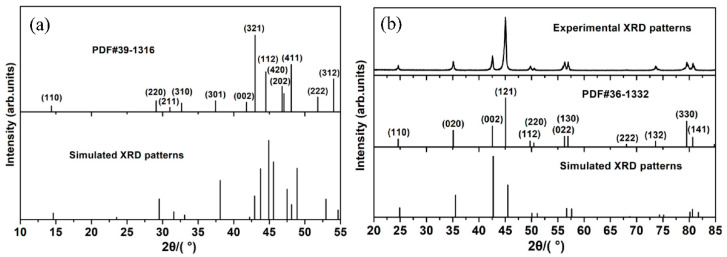
(**a**) Polycrystalline XRD patterns of (**a**) Fe_3_B and (**b**) Fe_2_B.

The equilibrium cell parameters of Ni_3_P-type Fe_3_B and its alloyed counterparts, together with some available experimental and calculated results, are presented in Table 1. The data of Fe_3_B show that the maximum deviations of lattice constants and cell volume between the calculated values and the previous results [28,41,42] are less than 2% and 4%, respectively, suggesting that our calculation method is credible. Note that small lattice distortion was triggered by alloying elements in all alloyed Fe_3_B, which is related to the differences of atomic radius and electronegativity between alloying elements and Fe. Figure 2 shows the atomic radius of alloying elements and the cell volume of all studied compounds. As can be seen, the variation trends of cell volume are almost identical to that of the atomic radius of alloying elements, implying that the difference of atomic radius dominates the lattice distortion. In addition, it can be found from Figure 2 that the cell volumes of the compounds are different from each other when the alloying elements occupy a different Fe site. This is mainly attributed to the different neighboring environments of different Fe sites. Previous research [29] reported that the Fe1, Fe2 and Fe3 have 12, 10 and 10 iron neighbors, respectively, and have 2, 4 and 3 boron neighbors, respectively. However, the present results demonstrate that the Fe3 has 11 iron neighbors and 2 boron neighbors. It is well known that Fe_3_B is a metastable phase, and thus it is relatively difficult to produce it, and further to obtain high-quality polycrystalline X-ray diffraction (XRD) data. The existing XRD data on Fe_3_B, PDF#39-1316, is marked with “?”, implying its lower reliability. Therefore, we simulated the XRD data of Fe_3_B based on the optimized crystal structure, and the pattern is given in Figure 3a. It can be found that the pattern of PDF#39-1316 is roughly consistent with the simulated one, especially at lower angles. Figure 3b shows the three patterns of Fe_2_B from the experiment [43], high-quality PDF card data and the simulation based on optimized crystal structure. They are in good agreement with each other, indicating that the simulated result is reliable. Thus, it can be inferred that the simulated XRD data of Fe_3_B is probably more credible than that of PDF#39-1316.

### 3.2. Thermodynamic Stability and Site Preference of Alloying Elements

In order to analyze the thermodynamic stability of alloyed Fe_3_B and the site preference of alloying elements, formation enthalpy (E_for_) and cohesive energy (E_coh_) were calculated according to the following equations:

E_for_ ((Fe_1−x_,M_x_)_3_B) = (E_total_ ((Fe_1−x_,M_x_)_3_B, cell) − 3n(1−x)E_cry_ (Fe) − 3nxE_cry_ (M) − nE_cry_ (B))/n (1)

E_coh_ ((Fe_1−x_,M_x_)_3_B) = (E_total_ ((Fe_1−x_,M_x_)_3_B, cell) − 3n(1−x)E_iso_ (Fe) − 3nxE_iso_(M) − nE_iso_ (B))/n (2)

In the equations, n is the number of Fe_2_B per unit cell, and E_total_ ((Fe_1−x_,M_x_)_3_B, cell) denotes the total energy of (Fe_1−x_,M_x_)_3_B per unit cell. E_cry_ (Fe), E_cry_ (B) and E_cry_ (M) are the energy of one atom for simple substance bcc Fe, α-B and M (bcc (V, Cr, Mo, W and Nb); hcp (Co and Ti) and fcc (Ni and Cu) and α-Mn), respectively. Finally, E_iso_ (Fe), E_iso_ (B) and E_iso_(M) are the energy of one isolated atom for Fe, B and M (Ti, V, Cr, Mn, Co, Ni, Cu, Mo, W and Nb), respectively.

**Figure 4:**
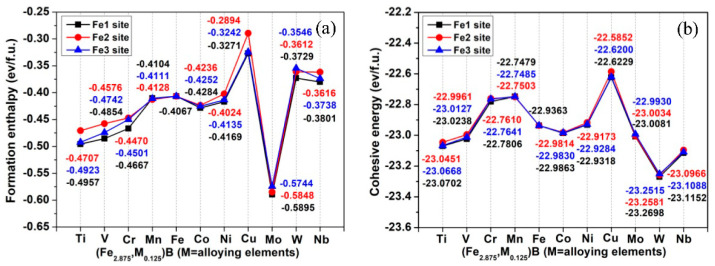
(**a**) Formation enthalpy and (**b**) cohesive energy of Fe_3_B and alloyed Fe_3_B.

The calculated formation enthalpy and cohesive energy of Ni_3_P-type Fe_3_B and its alloyed counterparts are shown in Figure 4. The results suggest that all studied compounds are thermodynamically stable, as their formation enthalpy and cohesive energy are negative. The E_c_ value of Fe_3_B is consistent with previously calculated results (−22.732 eV/f.u. [28] and −22.576 eV/f.u. [29]), and its E_for_ value is comparable with previously theoretical values (−0.864 eV/f.u. [28] and −0.880 eV/f.u. [44]). Although the formation enthalpies of (Fe_2.875_,Cu_0.125_)B, (Fe_2.875_,W_0.125_)B and (Fe_2.875_,Nb_0.125_)B are negative, Cu, W and Nb cannot easily enter the Fe_3_B lattice to replace the Fe atom since their formation enthalpies are greater than that of Fe_3_B. Similarly, Ni is also unable to easily substitute the Fe atom of the Fe2 site. (Fe_2.875_,Mo_0.125_)B has the lowest formation enthalpy, which means that it is easier to fabricate than the others. From the view of cohesive energy, (Fe_2.875_,W_0.125_)B is the most stable compound among those considered. The site preference of alloying elements is also judged by formation enthalpy and cohesive energy; namely, for the (Fe_2.875_,M_0.125_)B in which the M atom occupies the Fe1, Fe2 and Fe3 site, respectively, the lower energy denotes that the M atom prefers to occupy this site. Hence, Figure 4 indicates that all alloying elements prefer to occupy Fe1, Fe3 and Fe2 sites in turn except for Mn, Mo and W. Furthermore, Mn prefers to occupy Fe2, Fe3 and Fe1 sites in turn, while for Mo and W the order is Fe1, Fe2 and Fe3. It is necessary to emphasize that, apart from Mn, all the alloying elements prefer to enter the Fe1 site in the Ni_3_P-type Fe_3_B.

### 3.3. Electronic and Magnetic Properties

**Figure 5:**
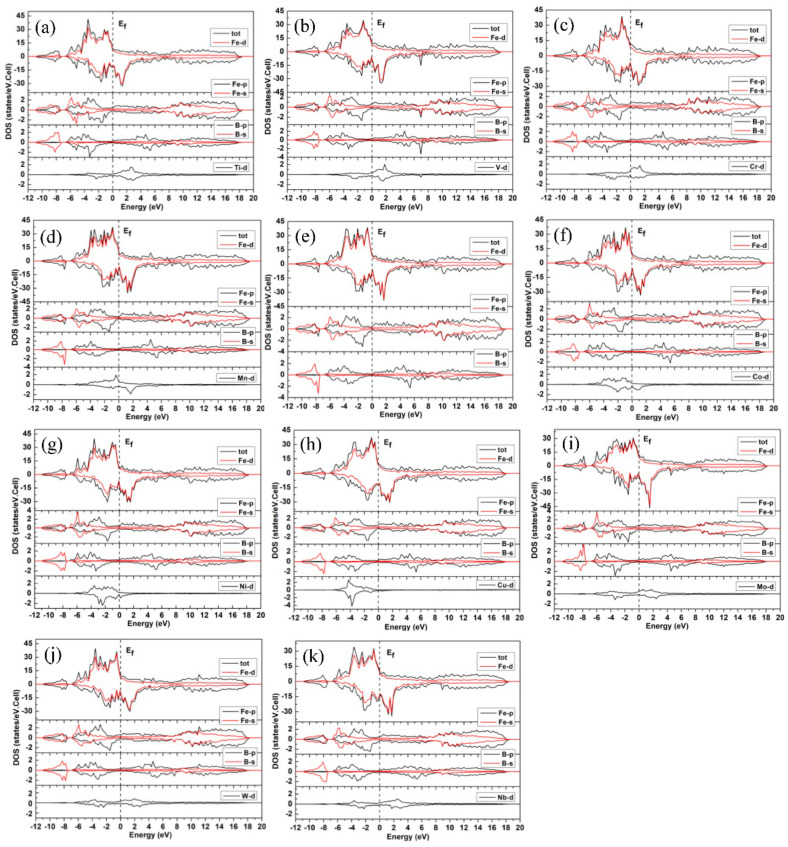
Total density of states (TDOSs) and partial density of states (PDOSs) of (**a**) (Fe_2.875_,Ti_0.125_)B, (**b**) (Fe_2.875_,V_0.125_)B, (**c**) (Fe_2.875_,Cr_0.125_)B, (**d**) (Fe_2.875_,Mn_0.125_)B, (**e**) Fe_3_B, (**f**) (Fe_2.875_,Co_0.125_)B, (**g**) (Fe_2.875_,Ni_0.125_)B, (**h**) (Fe_2.875_,Cu_0.125_)B, (**i**) (Fe_2.875_,Mo_0.125_)B, (**j**) (Fe_2.875_,W_0.125_)B and (**k**) (Fe_2.875_,Nb_0.125_)B.

To understand the bonding characteristics and magnetic properties of Fe_3_B and alloyed Fe_3_B, their spin-polarized total densities of states (TDOSs) and partial densities of states (PDOSs) were calculated, and the results are exhibited in Figure 5. Since Mn preferentially occupies the Fe2 site, whereas the others are more likely to occupy the Fe1 site, the DOS calculations for all alloying elements except for Mn are based on the model that the M atom resides in the Fe1 site; similarly, for Mn the calculations are based on the Mn atom preferentially occupying the Fe2 site. Figure 5 shows that all DOSs have a similar shape, suggesting that the compounds have similar bonding and magnetic characteristics. The absence of an energy gap at Fermi level (E_f_) indicates that a metallic bond should be contained in all compounds. In addition, it is easy to see that the TDOSs are greatly dominated by the Fe-d states. From the lower energy level to the higher energy level, all TDOSs and PDOSs of Fe and B are composed of the lower valence band, the upper valence band and unoccupied conduction band, in which there is an energy gap between the lower valence band and the upper valence band. Moreover, the lower valence band of TDOSs primarily consists of Fe-s, Fe-p and B-s states, whereas the upper valence band is mostly determined by the Fe-d states; additionally, a small number of Fe-p, Fe-s, B-p and M-d states can be observed. Due to the negligible contribution from M-s and M-p states, only the M-d states are presented in Figure 5. As can be seen, for 3d transition metals, more and more M-d states appear in the upper valence band with increasing valences, which is in good accord with the case of alloyed Fe_2_B [45]. The PDOSs reveal that there is a strong hybridization between the Fe-d states and the B-p states in the energy range of about −6 eV to −3 eV, which implies that all the compounds possess strong Fe-B covalent bonds. Moreover, the large overlaps between the Fe-d states and the Fe-s, Fe-p states suggest the existence of Fe-Fe covalent bonds.

**Figure 6:**
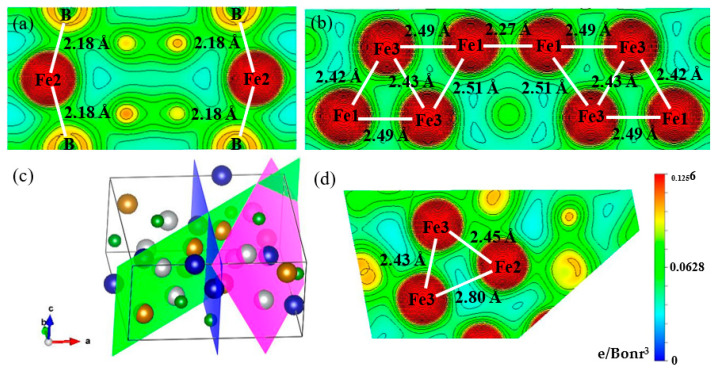
(**a**) Fe-B bonds with bond lengths of 2.18 Å; (**b**) Fe-Fe bonds with bond lengths of 2.27 Å, 2.42 Å, 2.43 Å, 2.51 Å and 2.49 Å; (**c**) crystal structure of Fe_3_B and (**d**) Fe-Fe bonds with bond lengths of 2.43 Å, 2.45 Å and 2.80 Å.

To gain insight into bonding nature, we checked the nearest-neighbor table of atoms, which was calculated on the basis of the optimized Fe_3_B crystal structure. The type of bond and their bond length are summarized in Table 2, and the visualized Fe-B and Fe-Fe bonds with typical bond length are presented by charge-density plots in Figure 6. From Table 2, four Fe-B bond lengths can be found: 2.12 Å (Fe3-B bond), 2.15 Å (Fe2-B bond), 2.16 Å (Fe1-B bond and Fe2-B bond) and 2.18 Å (Fe2-B bond). Figure 6a gives a Fe2-B bond of 2.18 Å, clearly demonstrating that this bond is covalent due to the appearance of elongated contours between the Fe2 atom and the B atom. Thus, it can be concluded that all Fe-B bonds are covalent, and the Fe3-B bond has the largest bond energy because it has the shortest bond length. The Fe-Fe bonds have many more bond lengths because the quantity of Fe atoms is three times that of B atoms. Moreover, as can be seen from Figure 6, the Fe-Fe bonds with bond lengths of 2.27 Å, 2.42 Å and 2.43 Å are covalent, whereas the Fe-Fe bonds with bond lengths of 2.45 Å, 2.49 Å, 2.51 Å and 2.80 Å are metallic bonds. Therefore, it is reasonable to deduce that the Fe-Fe bonds are metallic bonds when their bond lengths are larger than 2.43 Å. It can thus be concluded that the Fe_3_B contains Fe-B covalent bonds, Fe-Fe covalent bonds and Fe-Fe metallic bonds. As mentioned above, the DOSs of alloyed Fe_3_B are similar to those of the Fe_3_B. In other words, all alloyed Fe_3_B is mainly composed of Fe-B covalent bonds, Fe-Fe covalent bonds and Fe-Fe metallic bonds. Additionally, due to the addition of alloying element M, M-Fe bonds and M-B bonds can also be observed in the alloyed Fe_3_B. Moreover, the bonding strength of the other bonds is affected by the M atoms, revealed by the nearest-neighbor table of atoms for the alloyed Fe_3_B. These facts mean that it is very difficult to obtain the rule of the effect of alloying elements on the bonding behaviors of Fe_3_B. Therefore, we did not carry out a further analysis about the bonding behaviors of alloyed Fe_3_B.

**Table 2:** Types of bond and their bond lengths in Ni_3_P-type Fe_3_B.

Type of Bond	Fe-B			Fe-Fe						B-B
	Fe1-B	Fe2-B	Fe3-B	Fe1-Fe1	Fe2-Fe2	Fe3-Fe3	Fe1-Fe2	Fe1-Fe3	Fe2-Fe3	B-B
Bond length (Å)	2.16	2.15 2.16 2.18	2.12	2.27 2.62 2.67	2.43 2.72	2.43	2.61	2.42 2.49 2.51 2.67	2.45 2.80	-

**Figure 7:**
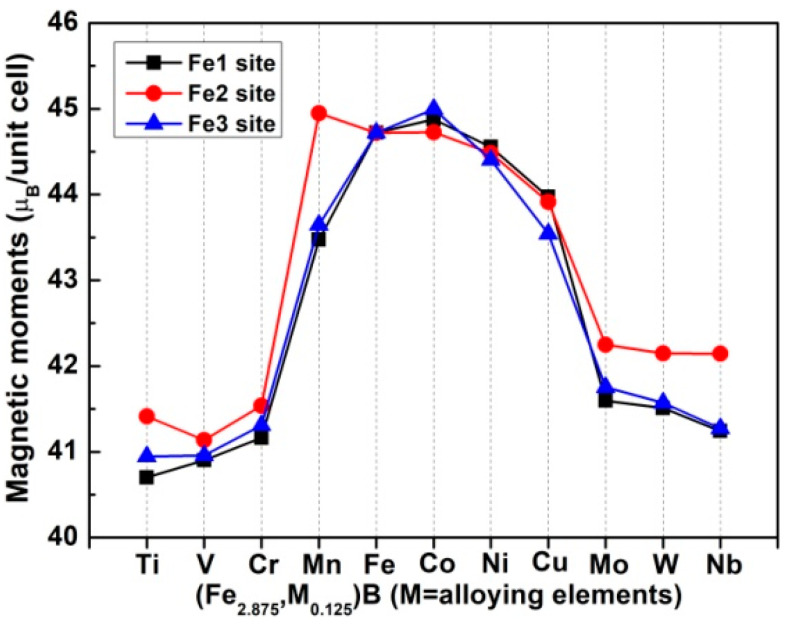
Magnetic moments of unit cells for Fe_3_B and alloyed Fe_3_B.

Additionally, as can be seen from Figure 5, all the TDOSs are mainly composed of two large majority-spin and minority-spin DOS peaks. Moreover, it is interesting to note that one of the minority-spin DOS peaks is located at the unoccupied conduction band, indicating that the majority-spin electrons are greater in number than the minority-spin electrons. In other words, all these compounds are magnetic. Moreover, their magnetism is significantly affected by Fe-d states. Here, in order to quantitatively analyze the effects of alloying elements and their site preference on the magnetic properties of Fe_3_B, we calculated the magnetic moments (Ms) values of unit cells, and the Ms values of different elements and the interstices of unit cells. For the calculations, the default Wigner–Seitz radii were employed, and only spin magnetic moments were considered because orbital Ms is very small. The results are presented in Table 3. It can be found that for Fe_3_B the calculated Ms of Fe is 1.921 μ_B_, which is consistent with previously obtained theoretical values 2.02 μ_B_ [28] and 1.99 μ_B_ [29]. Additionally, the Ms values of Fe1 (2.00 μ_B_), Fe2 (1.80 μ_B_) and Fe3 (1.97 μ_B_) match well with the previous results (Fe1 (2.14 μ_B_), Fe2 (1.89 μ_B_) and Fe3 (1.95 μ_B_)) [29]. It is necessary to point out that although the magnetic properties of some borides, such as Fe_3_B [23], (Fe_x_,Cr_1__−__x_)_3_B [23], (Fe_x_,Mn_1__−__x_)_3_B [12] and (Fe_x_,Co_1__−__x_)_3_B [11], have been measured by experiments, the crystal structures of these borides were not carefully and clearly distinguished. Thus, these experimental data will be discussed in a following work about the alloyed Fe_3_B with Ti_3_P-type structure, since according to the calculated results of the present work and the next work, their crystal structures can probably be distinguished. From the Ms values, it can determined that Fe_3_B, (Fe_2.875_,Mn_0.125_)B, (Fe_2.875_,Co_0.125_)B, (Fe_2.875_,Ni_0.125_)B and (Fe_2.875_,Cu_0.125_)B are ferromagnetic compounds, whereas the others are ferrimagnetic because the Ms values of Ti, V, Cr, Mo, W and Nb elements are negative. In addition, the B element reduces the Ms values of unit cells for all compounds even though its Ms value is small. Mn and Co have larger Ms values, especially when Mn occupies the Fe2 site. Figure 7 gives the Ms values of unit cells for Fe_3_B and alloyed Fe_3_B.

Overall, the Ms values of unit cells first increase, and then decrease, and only Mn and Co enhance the Ms of Fe_3_B. Regardless of which Fe site the Co occupies, the Ms values of (Fe_2.875_,Co_0.125_)B are larger those that of Fe_3_B. However, for the (Fe_2.875_,Mn_0.125_)B, only when Mn resides in the Fe2 site is its Ms value larger than that of Fe_3_B. The largest Ms values for Fe1, Fe2 and Fe3 sites are 44.877 μ_B_ ((Fe_2.875_,Co_0.125_)B), 44.948 μ_B_ ((Fe_2.875_,Mn_0.125_)B) and 44.996 μ_B_ ((Fe_2.875_,Co_0.125_)B), respectively. Combined with the stability of the compounds and the cost of production, it can be concluded that among all the studied alloying elements, Mn is the most favorable candidate for improving the magnetic properties of Fe_3_B.

## 4. Conclusions

We systematically investigated the effect of alloying elements on the stability, electronic structure and magnetism of Ni_3_P-type Fe_3_B, as well as the site preference of alloying elements, via first-principles density functional calculations, resulting in the following conclusions.

(1) Negative formation enthalpy and cohesive energy suggest that all studied compounds are thermodynamically stable, while Cu, W and Nb do not easily enter the Fe_3_B lattice. The lowest formation enthalpy implies that the (Fe_2.875_,Mo_0.125_)B can be produced more easily, whereas the lowest cohesive energy means that the (Fe_2.875_,W_0.125_)B is the most stable compound.
(2) Mn preferentially resides in the Fe2 site, whereas the others are more likely to occupy the Fe1 site. The Fe3 site is the second most popular site for most alloying elements.
(3) The DOSs of both Fe_3_B and alloyed Fe_3_B are dominated by Fe-d states, and the plots of charge density indicate that all the compounds mainly consist of Fe-B covalent bond, Fe-Fe covalent bond and Fe-Fe metallic bond.
(4) The Fe_3_B, (Fe_2.875_,Mn_0.125_)B, (Fe_2.875_,Co_0.125_)B, (Fe_2.875_,Ni_0.125_)B and (Fe_2.875_,Cu_0.125_)B are ferromagnetic compounds, whereas the others are ferrimagnetic compounds since the Ms values of Ti, V, Cr, Mo, W and Nb elements are negative. Although both Mn and Co can increase the magnetic moments of Fe_3_B, and although the maximum Ms values of (Fe_2.875_,Co_0.125_)B are slightly larger than those of (Fe_2.875_,Mn_0.125_)B, considering the cost of production, Mn is the more favorable candidate for improving the magnetic properties of Fe_3_B.

## Figures and Tables

**Table 1:** Cell parameters of Ni_3_P-type Fe_3_B and its alloyed counterparts.

Model	Cell Parameters						
	a (Å)	b (Å)	c (Å)	α (°)	β (°)	γ (°)	Volume (Å^3^)
Fe_3_B in this work	8.5538	8.5538	4.2106	90.0000	90.0000	90.0000	308.0780
Exp. in Ref. [35]	8.6470	8.6470	4.2820	90.0000	90.0000	90.0000	320.1677
Exp. in Ref. [37]	8.6300	8.6300	4.2900	90.0000	90.0000	90.0000	319.5059
Cal. in Ref. [24]	8.5510	8.5510	4.2400	90.0000	90.0000	90.0000	310.0271
**Fe1 Site**							
(Fe_2.875_,Ti_0.125_)B	8.5858	8.5860	4.2227	89.9012	89.9010	90.2751	311.2846
(Fe_2.875_,V_0.125_)B	8.5687	8.5689	4.2076	89.9141	89.9139	90.1963	308.9377
(Fe_2.875_,Cr_0.125_)B	8.5639	8.5641	4.1976	89.9331	89.9330	90.0826	307.8600
(Fe_2.875_,Mn_0.125_)B	8.5489	8.5489	4.2068	89.9763	89.9765	90.0448	307.4465
(Fe_2.875_,Co_0.125_)B	8.5580	8.5581	4.2049	90.0047	90.0044	89.9087	307.9688
(Fe_2.875_,Ni_0.125_)B	8.5648	8.5650	4.2029	90.0067	90.0058	89.8050	308.3100
(Fe_2.875_,Cu_0.125_)B	8.5755	8.5759	4.2072	89.9691	89.9684	89.8815	309.4071
(Fe_2.875_,Mo_0.125_)B	8.5991	8.5993	4.2237	89.9771	89.9764	90.0708	312.3262
(Fe_2.875_,W_0.125_)B	8.6001	8.6012	4.2255	89.9583	89.9576	90.0044	312.5921
(Fe_2.875_,Nb_0.125_)B	8.6182	8.6184	4.2370	89.9576	89.9572	90.2028	314.7009
**Fe2 Site**							
(Fe_2.875_,Ti_0.125_)B	8.5702	8.5750	4.2465	89.9251	90.0008	90.0011	312.0747
(Fe_2.875_,V_0.125_)B	8.5409	8.5763	4.2232	90.0730	90.0005	90.0000	309.3454
(Fe_2.875_,Cr_0.125_)B	8.5430	8.5635	4.2115	90.1504	90.0000	89.9997	308.1045
(Fe_2.875_,Mn_0.125_)B	8.5622	8.5526	4.2161	89.9576	89.9999	89.9998	308.7394
(Fe_2.875_,Co_0.125_)B	8.5592	8.5465	4.2089	90.0519	89.9999	90.0000	307.8827
(Fe_2.875_,Ni_0.125_)B	8.5645	8.5489	4.2114	90.1249	90.0000	89.9996	308.3460
(Fe_2.875_,Cu_0.125_)B	8.5650	8.5594	4.2206	90.1357	90.0001	89.9995	309.4180
(Fe_2.875_,Mo_0.125_)B	8.6100	8.5792	4.2427	90.2233	90.0010	90.0003	313.3920
(Fe_2.875_,W_0.125_)B	8.6098	8.5804	4.2446	90.2497	90.0010	90.0001	313.5696
(Fe_2.875_,Nb_0.125_)B	8.6271	8.5901	4.2643	90.0921	90.0015	90.0013	316.0192
**Fe3 Site**							
(Fe_2.875_,Ti_0.125_)B	8.5878	8.5879	4.2215	89.8791	89.8789	90.1831	311.3383
(Fe_2.875_,V_0.125_)B	8.5659	8.5660	4.2101	89.8952	89.8955	90.1594	308.9142
(Fe_2.875_,Cr_0.125_)B	8.5527	8.5528	4.2098	89.9875	89.9880	90.1606	307.9398
(Fe_2.875_,Mn_0.125_)B	8.5533	8.5533	4.2069	89.9555	89.9556	90.0387	307.7746
(Fe_2.875_,Co_0.125_)B	8.5464	8.5466	4.2171	90.0956	90.0958	89.9987	308.0246
(Fe_2.875_,Ni_0.125_)B	8.5422	8.5423	4.2239	90.0536	90.0527	89.9958	308.2164
(Fe_2.875_,Cu_0.125_)B	8.5509	8.5509	4.2264	90.0174	90.0159	90.0734	309.0206
(Fe_2.875_,Mo_0.125_)B	8.5952	8.5955	4.2318	89.9133	89.9140	90.1358	312.6414
(Fe_2.875_,W_0.125_)B	8.5968	8.5971	4.2331	89.8960	89.8963	90.1204	312.8589
(Fe_2.875_,Nb_0.125_)B	8.6245	8.6247	4.2337	89.9020	89.9018	90.1331	314.9125

**Table 3:** Average magnetic moments of Fe atom, B atom, alloying elements M, interstitial region and unit cell for Fe_3_B and alloyed Fe_3_B.

Model	Magnetic Moments in μ_B_				
	Fe	B	M	Interstice	Unit Cell
This work	1.921	−0.157	-	−0.123	44.719
Cal. in Ref. [24]	2.02	-	-	-	-
Cal. in Ref. [25]	1.99	-	-	-	-
**Fe1 Site**					
(Fe_2.875_,Ti_0.125_)B	1.855	−0.148	−0.564	−0.212	40.701
(Fe_2.875_,V_0.125_)B	1.873	−0.144	−0.853	−0.184	40.901
(Fe_2.875_,Cr_0.125_)B	1.899	−0.141	−1.262	−0.121	41.163
(Fe_2.875_,Mn_0.125_)B	1.894	−0.151	1.221	−0.097	43.472
(Fe_2.875_,Co_0.125_)B	1.953	−0.154	1.319	−0.123	44.877
(Fe_2.875_,Ni_0.125_)B	1.971	−0.152	0.571	−0.145	44.554
(Fe_2.875_,Cu_0.125_)B	1.965	−0.151	0.129	−0.149	43.971
(Fe_2.875_,Mo_0.125_)B	1.884	−0.143	−0.461	−0.130	41.595
(Fe_2.875_,W_0.125_)B	1.878	−0.142	−0.397	−0.159	41.510
(Fe_2.875_,Nb_0.125_)B	1.873	−0.146	−0.520	−0.161	41.242
**Fe2 Site**					
(Fe_2.875_,Ti_0.125_)B	1.880	−0.145	−0.477	−0.193	41.413
(Fe_2.875_,V_0.125_)B	1.874	−0.138	−0.693	−0.171	41.135
(Fe_2.875_,Cr_0.125_)B	1.898	−0.136	−0.922	−0.107	41.537
(Fe_2.875_,Mn_0.125_)B	1.938	−0.157	1.746	−0.109	44.948
(Fe_2.875_,Co_0.125_)B	1.956	−0.153	1.065	−0.106	44.726
(Fe_2.875_,Ni_0.125_)B	1.974	−0.150	0.420	−0.125	44.484
(Fe_2.875_,Cu_0.125_)B	1.964	−0.151	0.069	−0.126	43.914
(Fe_2.875_,Mo_0.125_)B	1.904	−0.139	−0.309	−0.135	42.247
(Fe_2.875_,W_0.125_)B	1.898	−0.136	−0.271	−0.154	42.147
(Fe_2.875_,Nb_0.125_)B	1.905	−0.142	−0.382	−0.158	42.145
**Fe3 Site**					
(Fe_2.875_,Ti_0.125_)B	1.863	−0.146	−0.530	−0.194	40.946
(Fe_2.875_,V_0.125_)B	1.871	−0.142	−0.773	−0.163	40.957
(Fe_2.875_,Cr_0.125_)B	1.897	−0.140	−1.090	−0.116	41.310
(Fe_2.875_,Mn_0.125_)B	1.895	−0.152	1.371	−0.105	43.644
(Fe_2.875_,Co_0.125_)B	1.961	−0.156	1.268	−0.128	44.996
(Fe_2.875_,Ni_0.125_)B	1.970	−0.155	0.508	−0.164	44.405
(Fe_2.875_,Cu_0.125_)B	1.950	−0.156	0.097	−0.167	43.542
(Fe_2.875_,Mo_0.125_)B	1.887	−0.141	−0.391	−0.123	41.752
(Fe_2.875_,W_0.125_)B	1.878	−0.140	−0.354	−0.149	41.570
(Fe_2.875_,Nb_0.125_)B	1.872	−0.144	−0.48	−0.149	41.272

## Data Availability

Data available upon request.
